# Maculopapular Exanthema After the Second Dose of Evolocumab

**DOI:** 10.7759/cureus.15249

**Published:** 2021-05-26

**Authors:** Victoria Ghernautan, Masoud Amini, Issac Sachmechi

**Affiliations:** 1 Internal Medicine, Icahn School of Medicine at Mount Sinai, NYC Health+Hospitals/Queens, New York, USA; 2 Research, NYC Health+Hospitals/Queens, New York, USA

**Keywords:** evolocumab, hyperlipidemia, hypersensitivity reactions, adverse effect, maculopapular exanthema, drug rash, drug hypersensitivity reactions, monoclonal antibodies

## Abstract

Evolocumab is a relatively new monoclonal antibody designed to decrease low-density lipoproteins via the inhibition of proprotein convertase subtilisin/kexin type 9 (PCSK9). It is used alone or in combination with other lipid-lowering agents. Evolocumab was associated with adverse events of skin rashes in clinical trials. We describe a rare case of maculopapular exanthema in a female patient with hyperlipidemia, which was treated with evolocumab. The patient was a 60-year-old female with hyperlipidemia who experienced a maculopapular rash after she was administered the second dose of evolocumab subcutaneously. The rash occurred on her torso and upper extremities and was associated with pruritus and mild wheezing. The hypersensitivity reaction was treated with antihistamines and with the discontinuation of evolocumab. The skin eruption cleared within 10 days. In conclusion, medical professionals should be aware of evolocumab skin hypersensitivity reactions, which could demand the cessation of the evolocumab treatment.

## Introduction

Drug hypersensitivity reactions have various presentations such as transient and benign erythema, maculopapular exanthema (MPE), fixed drug eruption, as well as severe reactions such as Stevens-Johnson syndrome, toxic epidermal necrolysis, and drug reaction with eosinophilia and systemic symptoms (DRESS) [1,2]. MPE is a mild skin eruption and is characterized by generalized macular and/or papular rash [1,2]. We present the case of a 60-year-old female with hyperlipidemia who developed a hypersensitivity skin eruption manifested as MPE and associated wheezing after receiving the second dose of evolocumab, which resulted in the discontinuation of the medication.

## Case presentation

**Figure 1:**
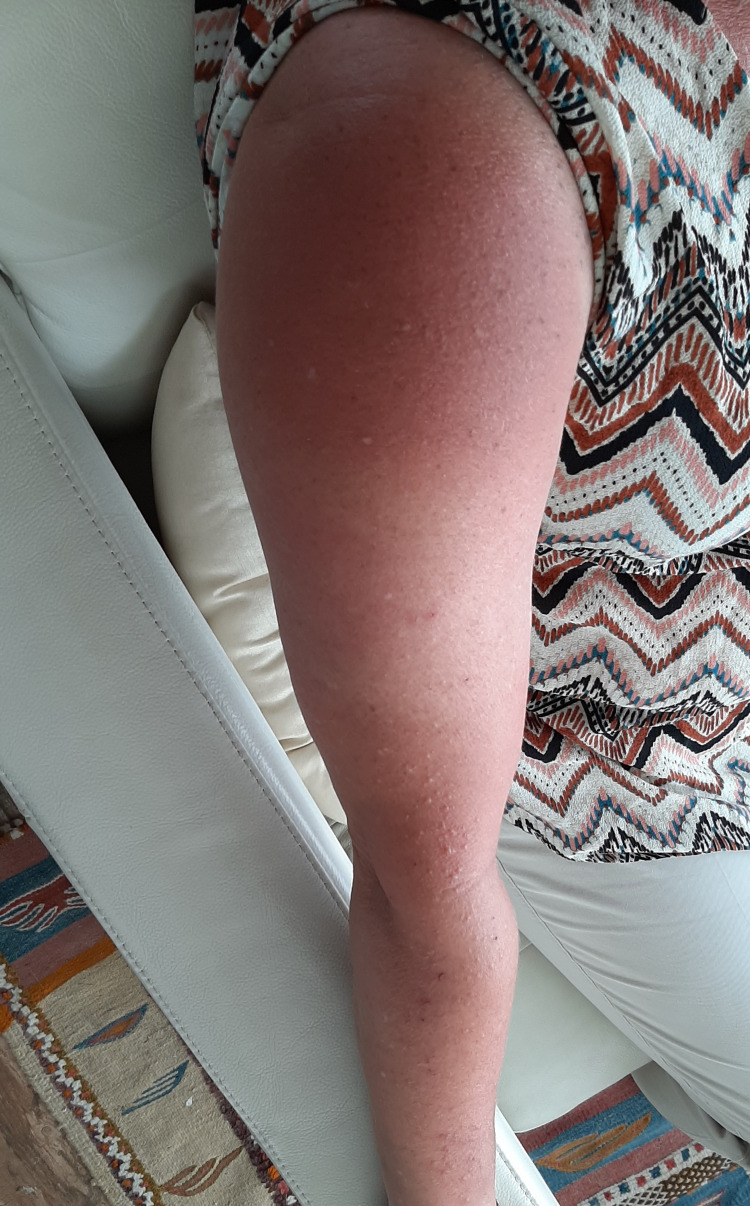
Maculopapular exanthema on the upper extremity secondary to evolocumab.

**Figure 2:**
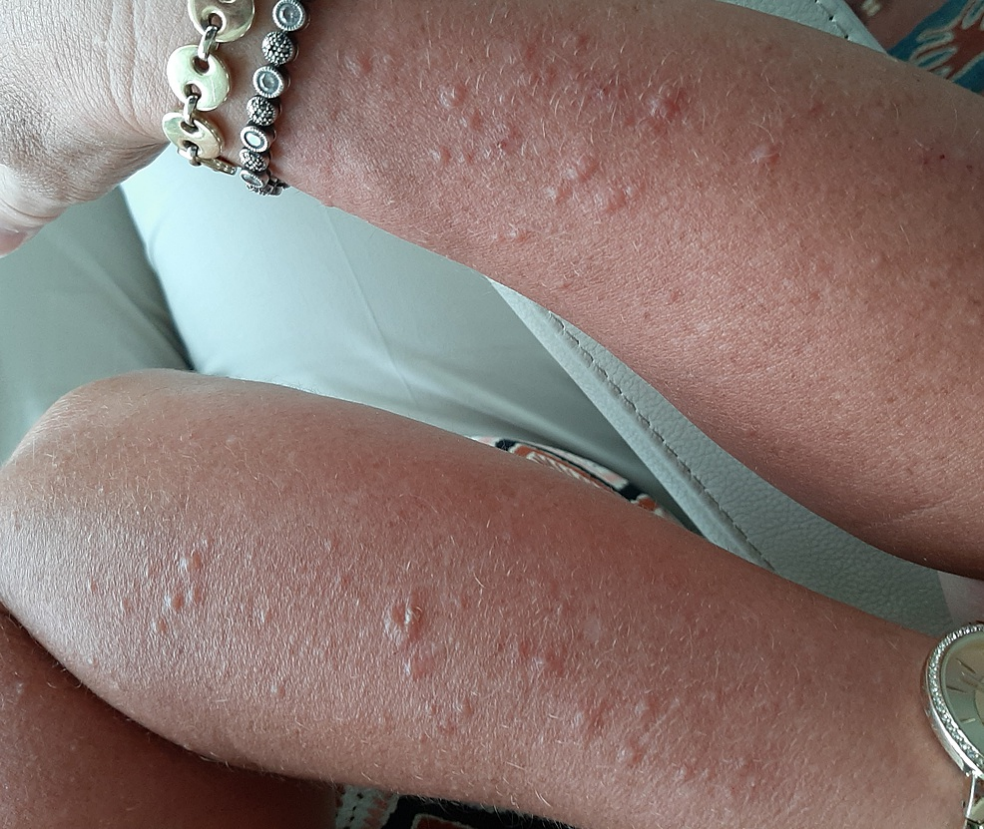
Papular rash on the dorsal aspect of the forearms secondary to evolocumab.

This is the case of a 60-year-old female with prediabetes, obesity, hypothyroidism, and hyperlipidemia, who was recently started on evolocumab. The patient was on statin therapy (pravastatin 40 mg daily, then switched to rosuvastatin 10 mg daily) for hyperlipidemia; however, it was discontinued due to the side effect of myalgia. Her lipid panel before starting the statins was not available, as she had different providers before joining our clinic. Evolocumab was started at the dose of 140 mg/ml subcutaneously every two weeks. The patient tolerated the first dose well. However, a day after the second evolocumab dose, the patient manifested MPE on the upper extremities, neck, and chest, associated with pruritus and mild wheezing (Figures 1, 2). There was no local reaction at the injection site, and the patient did not have any dyspnea, hypotension, or tongue swelling. The patient had not had any prior history of allergies or asthma. She was on levothyroxine 125 mcg daily for hypothyroidism and did not start new medications or had allergen exposure. The rash cleared within 10 days with oral and topical antihistamines. An inhaled steroid/long-acting β₂ adrenergic receptor agonist was given for wheezing. Evolocumab was discontinued, and the patient was prescribed bempedoic acid for hyperlipidemia.

## Discussion

Adverse drug reactions can affect 10% to 15% of patients taking medications, and the skin is involved in approximately 20% of cases [3]. Patients at risk of experiencing drug hypersensitivity reactions include those with a previous history of such reactions, patients on multiple drugs, and patients with concomitant diseases such as HIV, autoimmune disorders, and asthma [3]. Most cutaneous drug eruptions are delayed type IV hypersensitivity reactions. A sub-classification of this reaction type was proposed, as the traditional Gell and Coombs model does not include all the various presentations of hypersensitivity to medications. It is based on the specific cell, which becomes activated, monocytes (type IVa), eosinophils (type IVb), and neutrophils (type IVd) [1]. Cytotoxicity (type IVc) predominates in many drug reactions [1].

Drugs are foreign substances that are precepted as antigens that bind the T-cell receptors to induce immune responses [2]. This is especially valid for biological drugs since they have non-self proteins in their component, which can potentially promote immune-mediated side effects [4,5]. There are several differences in the structure, molecular weight, and technology of traditional drugs and biologics; therefore, the side effects of biologics are expected to have distinct mechanisms. Adverse effects of biologic drugs, such as hypersensitivity, are encountered less frequently compared to infusion reactions, although they share common features [5].

Evolocumab is a biological agent, a human monoclonal antibody (mAb) designed to decrease low-density lipoprotein (LDL) cholesterol. It works by inhibiting proprotein convertase subtilisin/kexin type 9 (PCSK9), which prevents it from degrading LDL receptors, leading to enhanced removal of LDL. It is the second PCSK9 inhibitor approved by the U.S. Food and Drug Administration (FDA) on August 27, 2015, for the treatment of hyperlipidemia in heterozygous and homozygous familial hypercholesterolemia and clinical atherosclerotic cardiovascular disease [6].

The safety of evolocumab was assessed in eight placebo-controlled trials and revealed several adverse events such as nasopharyngitis (10.5%), upper respiratory tract infections (9.3%), back pain (6.2%), and myalgias (4.0%). Rash and urticaria were encountered; however, their incidence was not reported [6]. According to the manufacturer, hypersensitivity reactions occurred in 0.4%-1% of patients receiving evolocumab [7].

In the OSLER-1 (Open Label Study of Long Term Evaluation Against LDL-C Trial) study, an open-label study, which evaluated the effectiveness and safety profile of evolocumab over a five-year period, hypersensitivity reactions were noticed in 10.2 % of patients in the first year of use. However, their incidence decreased over time by 5.6% of cases in the fourth year [8]. The GAUSS-3 (Goal Achievement After Utilizing an Anti-PCSK9 Antibody in Statin Intolerant Subjects-3) randomized clinical trial, which compared the efficacy and tolerability of evolocumab versus ezetimibe in patients with muscle-related statin intolerance, revealed the adverse events in the form of a rash in 3.4% of 145 patients receiving evolocumab. The skin eruptions, along with nausea, insomnia, and acid reflux were the least encountered side effects in that study [9]. There was a case published in 2019, which reported a rash mimicking atopic dermatitis associated with high-dose evolocumab therapy [10].

A large study, which published real-world data of evolocumab and alirocumab safety using a hospital registry and two pharmacovigilance databases, revealed influenza-like illness (27.3% and 28.6%, respectively) and myalgia (7.8% and 9.4%, respectively) to be among the most common side effects [11]. Myalgia was the main reason for discontinuation of both mAbs. Rash was reported in four (5.9%) of 68 patients [11]. In the randomized, controlled, open-label DE LAVAL study, one patient discontinued the evolocumab treatment due to worsening rash on the face and neck, which was attributed to evolocumab [12].

Our patient developed a pruritic MPE a day after she received the second dose of evolocumab, which prompted us to suspect a cutaneous drug reaction. This case adds valuable knowledge about the adverse event of MPE secondary to evolocumab treatment.

## Conclusions

Clinicians should be vigilant regarding potential hypersensitivity reactions during evolocumab therapy. It could require immediate termination of evolocumab administration and subsequent application of alternative treatments. Our case urges providers to share their experience on unexpected drug reactions to contribute to the postmarketing surveillance of drug adverse effects.
